# Assessment of groundwater aquifer using geophysical and remote sensing data on the area of Central Sinai, Egypt

**DOI:** 10.1038/s41598-023-44737-9

**Published:** 2023-10-25

**Authors:** Sultan A. S. Araffa, Hamed G. Hamed, Alaa Nayef, Hassan S. Sabet, Mostafa M. AbuBakr, Mohamed El. Mebed

**Affiliations:** 1 National Research Institute of Astronomy and Geophysics (NRIAG), Helwan, Cairo, 11421 Egypt; 2 Egyptian Mineral Resources Authority (EMRA), Abbassia, Cairo, 11517 Egypt; 3 National Authority for Remote Sensing and Space Sciences, Cairo, Egypt; 4 Geology Department, Faculty of Science, Al-Azhar University, Cairo, 11884 Egypt

**Keywords:** Environmental sciences, Hydrology, Solid Earth sciences

## Abstract

The study aims to assess groundwater resources in Sinai's central area using remote sensing, geoelectric, and well-logging data, utilising techniques for modelling hydrogeological frameworks and evaluating desert regions' groundwater potential. Its utilized satellite image sources, soil maps, and geological maps to map the effects of various factors on groundwater potentiality recharge, dividing it into five zones. Eighteen deep VES stations were used to examine the upper part of the groundwater aquifer in Central Sinai, Egypt, comparing it with available borehole information (Well-1, and JICA-1) to establish subsurface geology and hydro-geology positioning. Borehole data, VES interpretation results, hydro-geophysical maps, and four geoelectrical cross-sections were used to visualize the rearward expansion of eight lithological units, groundwater-bearing sections, and aquifer-filled thicknesses. From interpretation data output reveal three zones with significant recharge and storage potential, including two groundwater aquifers. The shallow aquifer has a saturation thickness of the fractured limestone of 35–250 m, while the deep aquifer Nubian sandstone is detected at depths ranging from 660–1030 m. NW–SE and NE–SW faults likely recharge conduits connecting shallow and deep aquifers, providing sites with acceptable groundwater potential for living, agriculture, and development in Sinai.

## Introduction

**Figure 1:**
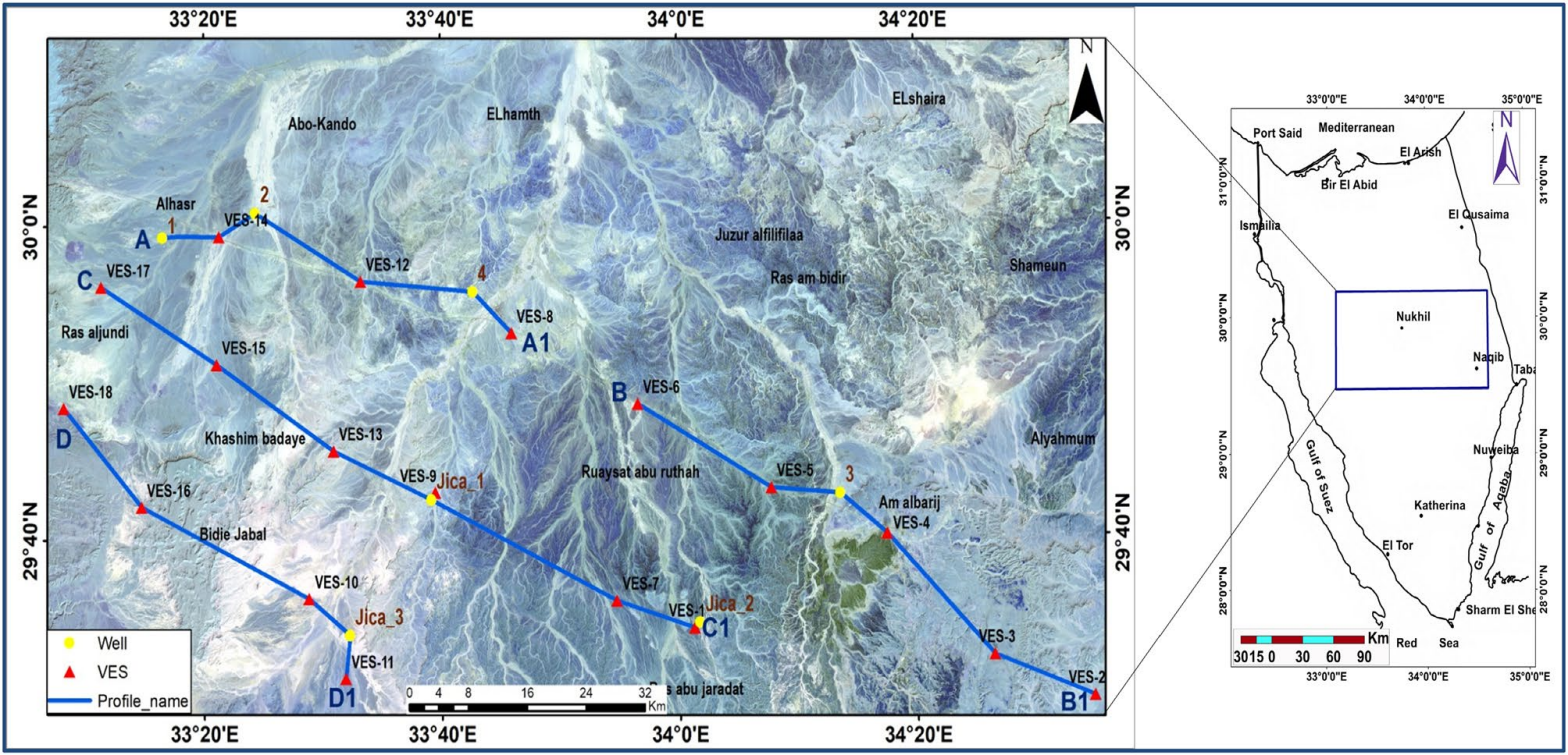
Landsat image and research area location map.

This paper aims to delineate groundwater accumulation by determining and investigating the influence of sub-basin physiographic features on groundwater recharge and storage. The researched area is mostly in the middle of Sinai, Egypt. They are located using latitudes and longitudes. The coordinates are 29°28′49″–30°11′44″ N and 33°05′34″–34°37′09″ E. It has an area of approximately 11,687 km^2^. According to the map of Fig. 1, it is located in the centre of Sinai, between the upper portion of the Gulfs of Suez, Aqaba, the external northeast border to Palestine, Al Hassana City, and Gebel Yelleg to the north. The geomorphology of the area is distinguished by high ridges such as Jebels Umm Ali, Abu Hamth, Khashm Budaya, Shameun, Sumar, Hitan, AD Dirsah, Abu Hajmiaat, Al Yahmum, and Al Thimid. Bedouins live in this area, although they have a water shortage for household use. As a result, the current work was used to delineate aquifers of groundwater, particularly deep aquifer Nubian sandstone. The majority of the study location is made up of lowlands and wadis, as well as Tertiary and Cretaceous deposits. Many geologic units, spanning from Cretaceous to Quaternary rocks, cover the area under study. It contains various valuable groundwater resources that can fund future development initiatives^1,2^. In order to ensure water availability, geophysical field evaluations determine the quantity of groundwater in Nubian Sandstone and drill locations for effective extraction. Geophysical explorations are a quick and inexpensive way to acquire knowledge about subsurface hydrogeology. Because of rapid improvements in computer science for the development of modelling techniques, geophysical techniques in groundwater estimating and water quantity assessment have expanded significantly during the past few years^3,4^. There are many hydro-geophysical procedures obtainable; electrical resistivity is a commonly employed method due to its inexpensive nature, ease of usage, and success in locations with strong differential resistivity, like that among weathered waste and bedrock^5^. Because hydrogeological factors like permeability and porosity may be connected with electrical resistivity values, geoelectrical approaches are especially useful for groundwater research. Geoelectrical processes have to do with determining the electrical resistivities of subsurface components to provide information on geological layers, structures, and groundwater occurrences^6–8^. Using deep VES stations of AB/2 up to 3000 m, geoelectrical resistivity is a promising tool for locating the freshwater quality aquifer (Nubian sandstone)^9–11^. Groundwater conditions and their occurrences may be supplied in addition to utilizing data from remote sensing, like imagery from satellites that can be analyzed using up-to-date programs^12^. Groundwater exploration is made possible by GIS and satellite remote sensing, especially in areas that are arid^13,14^. The processing of images generates valuable data that aids in understanding groundwater spatial distribution^15–17^. GIS is useful for defining groundwater potential areas because it can analyze and integrate multiple spatially distributed data sets with a variety of logical criteria. Several researches^12,13,18–23^ have successfully used GIS techniques to map groundwater availability zones. Gravity, well-log data, and geoelectrical resistivity data are used to determine the thickness, depth, and structural components of this aquifer. Earlier research has focused on the shallow aquifer, which contains brackish water to varying degrees. All of these tools could be combined to produce satisfying outcomes and a clear picture of geological formations. The gravity method delineates the subsurface of this aquifer's thickness, depth, and structural components structures that can be dissected by the sedimentary layer and upper surface of basement rocks. According to various geophysical investigations carried out there and elsewhere, including those by^9,24–26^, the studied area features deep groundwater wells. Subsurface structures, engineering geology, groundwater investigation, and ore mineral exploration can all be accomplished with geophysical methods^27–34^.

### Geologic setting

**Figure 2:**
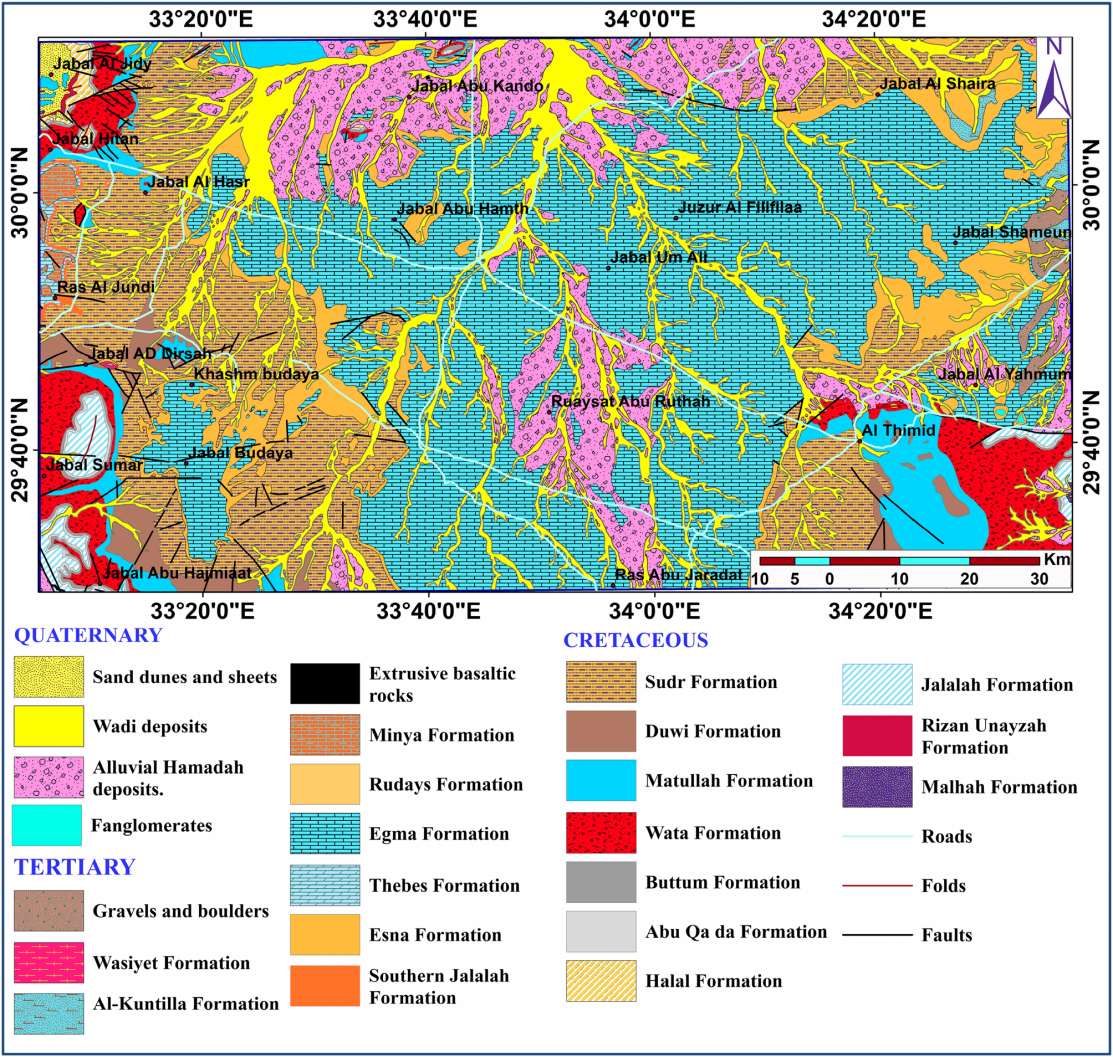
Geological map of the study area (modified after^41,43^).

**Figure 3:**
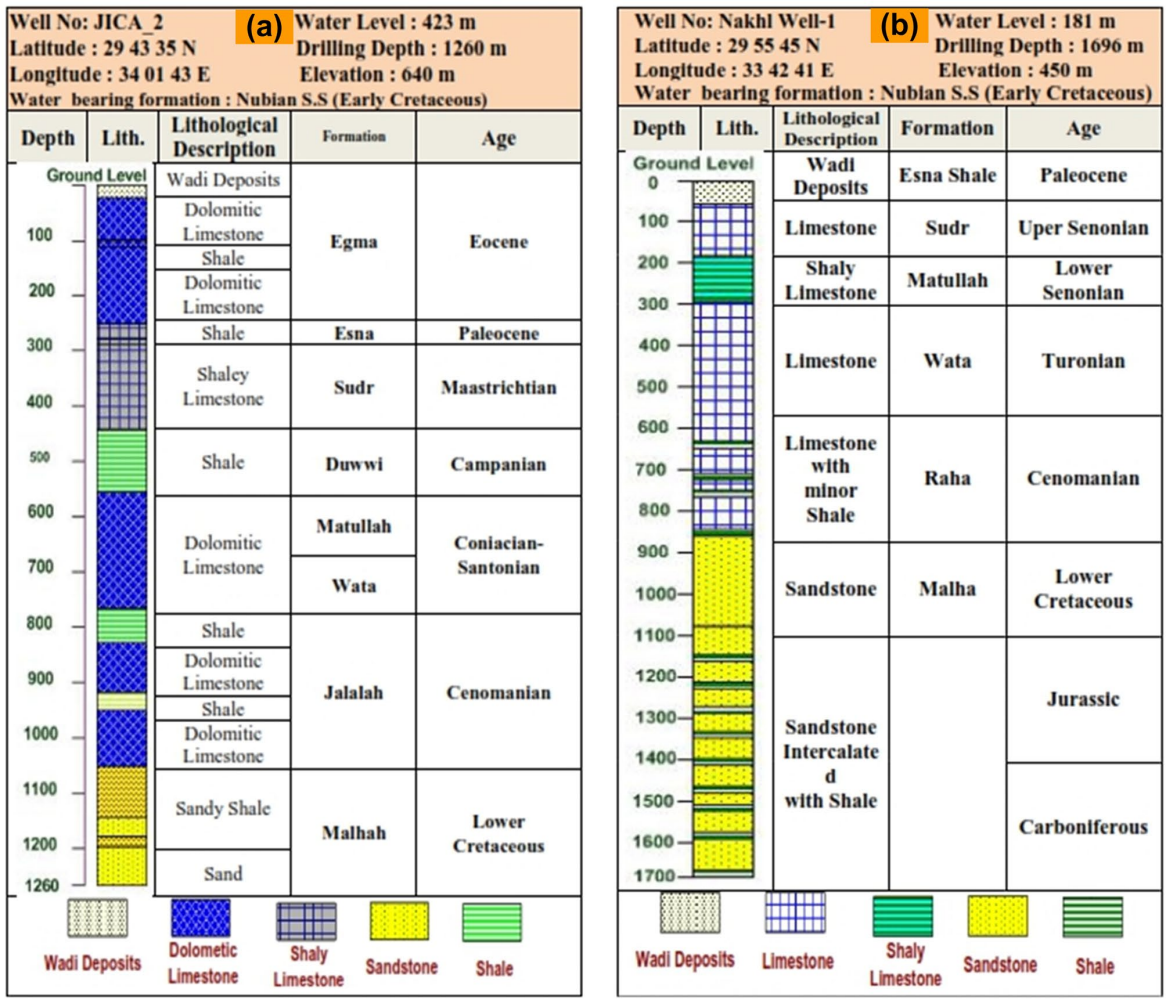
(**a**) Jica Well No. 2 lithostratigraphic sequence, (**b**) Nakhl Well-1 lithostratigraphic sequence.

The Sinai geology has a fascinating history that began at the end of the first quarter of the twentieth century. Works^35^ provided us with some excellent background material. Many authors, including^36–40^, have conducted various geomorphologic investigations in the area. Sinai's middle zone is separated into a number of geomorphic units, including the tableland area (El Tih-El Egma plateau), the dissected hilly area, inland depressions, coastal plains, and alluvial deposits (deltas) of the hydrographic basin. The age distribution of the majority of outcropping rock units in the research area ranges from upper Mesozoic to Cenozoic (Fig. 2). Sinai's geology map, produced by^41^ shows local surface geology. Quaternary deposits, including sand dunes, wadi deposits, and Hamadah deposits, dominate the study surface. These deposits have been identified throughout the majority of the lowlands in the research region, including Wadi El Arish. The surface and subsurface lithostratigraphic successions were characterized by geologic maps and wells acquired during the inquiry^42,43^. The full depth of the JICA-2 well is 1260 m, and the Nakhl well-1 has a total depth of 1700 m (Fig. 3a,b). Tertiary deposits are classified into stratigraphic units ranging in age from younger to older: Fanglomerates, Gravels and boulders, Wasiyt Formation (limestone with Nummulites desert and conglomerate bands at base), Al-Kuntilla Formation (claystone and limestone, followed upwards by gritty sandstone intercalated with calcareous claystone), Extrusive basaltic rocks, Minya Formation (white chalky in the upper part, greyish and yellowish white in the lower part), Rudays Formation (Marl and sandstone with fossiliferous carbonate beds), Egma Formation: Chalky limestone with flint and chert bands. Thebes Formation (Nummulitic limestone, dolomitic in the upper part), Esna Formation (Greenish marly shale and marly limestone rich in fossils), Southern Jalalah Formation (argillaceous limestone, intercalated with marl and sandy marl lenses). Cretaceous deposits were split into a number of stratigraphic units that ranged in age from younger to older: Sudr Formation (white to pale grey chalk, marly and shale bed), Duwi Formation (alternating clastic and carbonate layering with phosphatic intercalations and a chert band cap), Matullah Formation (Argillaceous limestone, marl and shale), Wata Formation (limestone and dolomitic, intercalated with sandstone and shale), Buttum Formation (Vari-colored shales alternating with crystalline gypsum and fine-grained sandstone), Abu Qa’da Formation (Carbonate rocks, shale, and marly-limestone), Halal Formation(dolomitic limestone, marl, and claystone), Jalalah Formation (marl, claystone, and limestone), Rizan Unayzah Formation (Fossiliferous limestone, sandstone, claystone beds). The area under study contains a substantial groundwater aquifer known as Nubian Sandstone, which includes fresh water at depths of (663 m) and (1030 m) at well JICA-2^42,43^, Malhah Formation (kaolinized sandstone embedded in mudstone and conglomerate) is a good example of this.

## Data and methods

### Remote sensing data

Recharge zones and groundwater potential were identified using Landsat-8, DEM, soil, lithological, and rainfall data. Data from boreholes were used to validate the results. Spatial analysis plots factors affecting groundwater potential (LULC, soil, lithology, rainfall, drainage density, lineament density, slope, and elevation) using a hierarchy technique to rank them based on relevance and how they affect groundwater recharge and potential. The study uses geospatial techniques to identify shallow groundwater zones using knowledge-based factor analysis (geology, slope, lineament, drainage density, rainfall, soil, and LULC). The investigation's area's remote sensing data was processed using image processing software, namely ENVI 5.3. ArcGIS 10.5 software was employed for Geographical Information Techniques. GIS hydrology tool determines drainage basin boundaries and density using SRTM-30m data. LULC was created using the LANDSAT-8 image. Several theme layers were created using the RS and GIS tools, as discussed in the following parts.

### Land use land cover (LULC)

**Figure 4:**
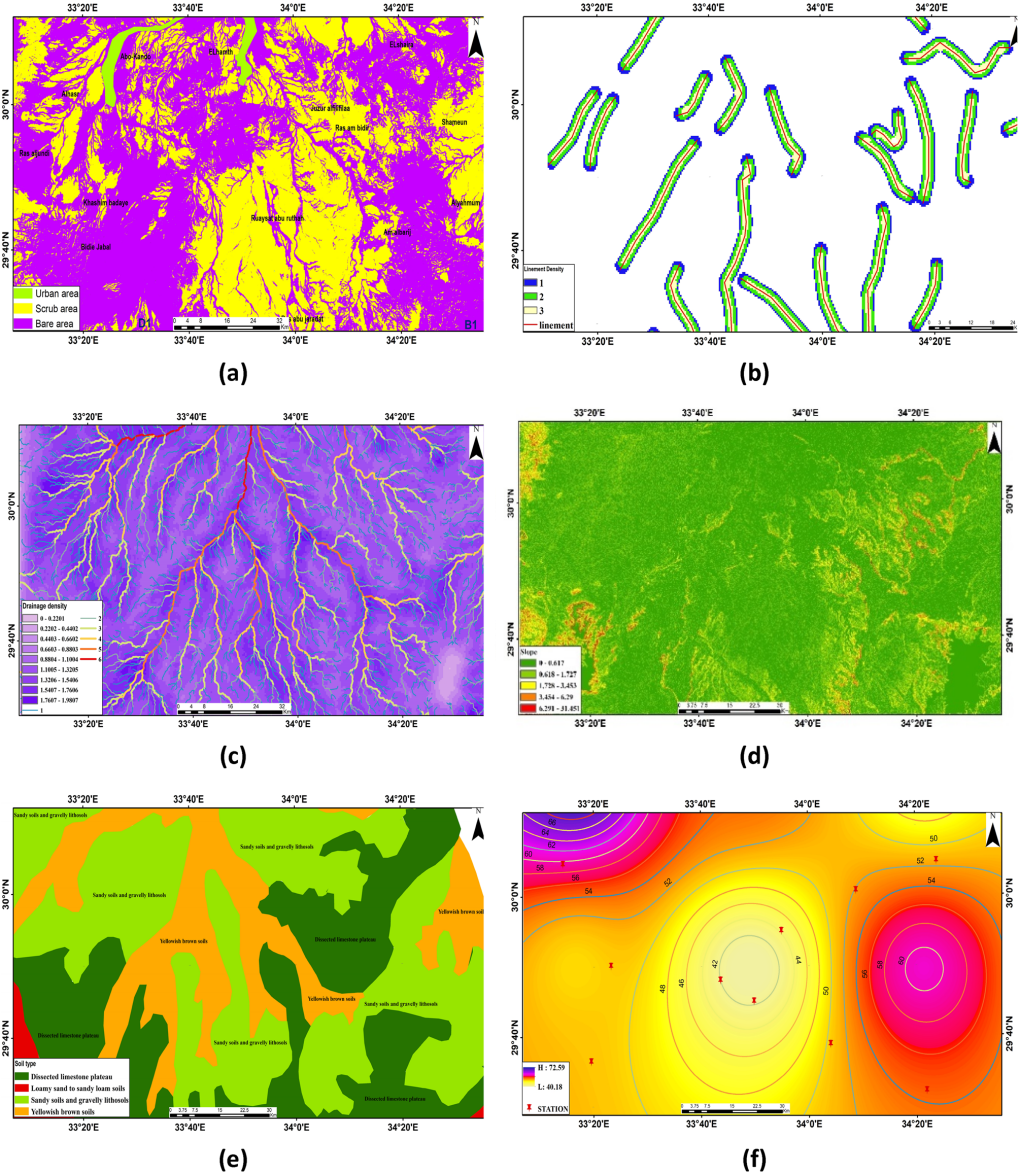
Thematic maps of the research area. (**a**) LULC classification. (**b**) Lineament buffer. (**c)** Drainage density. (**d**) The slope. (**e**) The soil types. (**f**) The rainfall rate.

LULC analyses infiltration rates, soil moisture, groundwater, and recharge, highlighting human activity's impact on ecological systems^44,45^. The majority of the study locations are bare land or scrubland, according to the LU/LC map in Fig. 4a, with some developed areas.

### Lineament

It differs from satellite imagery in that it usually has straight alignments. Lineaments are faults and cracks that cause secondary porosity and permeability to increase. DEM satellite data is used to extract the lines of this area. The lineament densities map, illustrated in Fig. 4b, was constructed using GIS software. Finding potential groundwater occurrence places can be done well by correlating structural elements such as fractures, joints, faults, and bedding planes for lineaments^46^. Based on a thorough analysis of the results, the data were divided into three groups: low (orange), moderate (green), high (blue), and extremely high (on the line with red colour). The density of lineaments is ranked based on their proximity and correlation with a geological map. It is found that as one moves away from the lineaments, the intensity of the groundwater potential decreases. Classes with a high density are given a heavy weight, whereas classes with a low density are given a lighter weight.

### Drainage density

The density of drainage has a considerable impact on groundwater availability and contamination^47^. Lithology impacts the drainage system, indicating infiltration rate; permeability and drainage density are inverse. Determining potential groundwater zones relies on drainage density, calculated by dividing stream lengths by basin area^21^. High drainage density weakens infiltration, affecting groundwater potential. Heavy infiltration, which increases groundwater potential, is indicated by low drainage density. For potential groundwater zonation, the low density received a high weight; whereas the high density received a low weight, Fig. 4c displays the research area's drainage density map.

### Slope

Slope significantly influences the groundwater potential zone, affecting infiltration and runoff rates. Moderate to steep slopes promote surface runoff, while low or near-level slopes promote robust infiltration and good groundwater recharge. A slope map was produced using SRTM elevation data^48,49^ and ArcGIS software. The slope map has been split into five major categories (Fig. 4d). Slopes that are thick and gentle are given larger weights. Low weight is offered for slopes that are steep and extremely steep.

### Soil

A key factor in identifying the potential groundwater occurrence zone is the soil. Calculating infiltration rate considers hydraulic properties and soil texture, with four main types: clay loam, clay, sandy loam, and sandy clay loam (Fig. 4e). One of the key elements influencing surface runoff and precipitation infiltration in the area is the soil texture. Sandy soil has a low runoff ratio and a high groundwater potential, in contrast to clay soil, which has a high runoff ratio and a very low groundwater potential. Sandy soil has a higher infiltration rate than clayey soil, which has the lowest ability for infiltration.

### Precipitation (rainfall distribution)

The hydrological cycle's principal source of water is rainfall. The rainfall pattern related to the outermost gradient affects runoff and penetration rate, revealing probable groundwater zones. IDW interpolation creates rainfall patterns based on highest and minimum values, which range from 40.18 to 72.59; the rainfall has been split into various groups. Infiltration is influenced by rainfall quantity and duration. High-intensity and short-duration rain impact infiltration less, while low-intensity and long-duration rain have a greater impact. High rainfall produces high weights, and oppositely. Figure 4f displays stations and rainfall interpolation map.

### Groundwater potential map

Many studies, including that of^14–16,18,48,50,51^ have used the Multi-Criteria Evaluation technique developed by^16^ to predict groundwater. The weighted overlay approach is used to weigh thematic layers based on expert knowledge. Several rasters are multiplied by their weight and combined^15,16,50,51^. Utilized ArcGIS weighted sum overlay strategy to create groundwater potential map using various layers mentioned above. Each raster layer was ranked according to exactly how much it was believed to contribute to groundwater infiltration and occurrences. Rainfall rate, slope, drainage density, lineaments, geologic map, soil type map, and LULC, for example, were assigned weights of 38%, 25%, 13%, 9%, 6%, 5%, and 3%, respectively, based on their importance in controlling groundwater recharging processes. Normalized weights for theme layers were determined by multiplying weights by total thematic layers^15,52^ (Table 1). Thematic layers are weighted based on infiltration qualities, and their degree is interpreted into GIS weights. Table 1 displays the significance of thematic layers in enhancing groundwater potential. Lower weight indicates reduced recharging capacity, calculated element weight as a proportion to potential recharge weight. The final GWPZ map is generated by multiplying the weights of thematic layers^50,52,53^.

1 $$ {\text{GWPZ }} = \, \sum {\text{Wi }} \times {\text{ CVi}} $$

where, GWPZ = Groundwater Potential zones, Wi = normalized thematic layer weight, CVi = capability value (normalized feature/subclass weight).

**Table 1:** The weights assigned to different thematic layers and classifications.

Criteria			
Thematic layer	Rank		Scale (1–9)
Rainfall	38	Geology	3
		Slope	3
		Drainage density	5
		LULC	5
		Lineament density	5
		Soil	7
Geology	25	Slope	3
		Drainage density	3
		LULC	5
		Lineament density	5
		Soil	5
Slope	13	Drainage density	1
		LULC	3
		Lineament density	3
		Soil	5
Drainage density	9	LULC	1
		Lineament density	2
		Soil	3
LULC	6	Lineament density	1
		Soil	3
Lineament	5	Soil	1
Soil	4	Intensity of rank	Definition
total	100	1	Equal importance
		3	Moderate
		5	strong
		7	Very important
		9	Extreme
		2, 4, 6, 8	Intermediate

**Figure 5:**
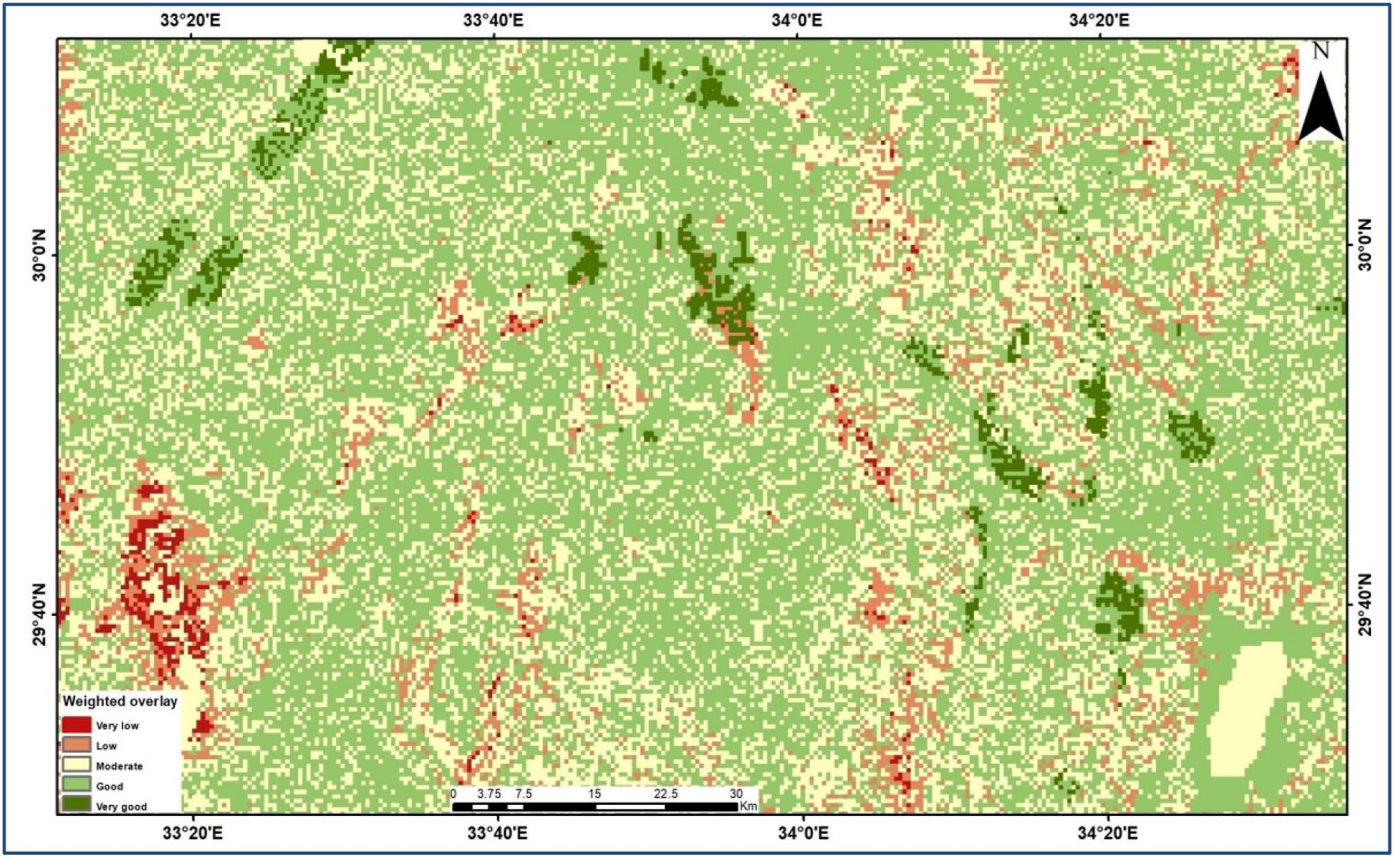
Map representing the potential groundwater zones.

Seven classifications were made for the GWPZ map that was created through the integration of all of the thematic maps, which vary from extremely low to exceptional potentiality (Fig. 5). Whereas the dark green areas have an excellent chance for groundwater potential, the red areas have a low potential for groundwater; in shallow aquifers (fractured limestone; second layer).

### Gravity data

#### Gravity data acquisition and interpretation

**Figure 6:**
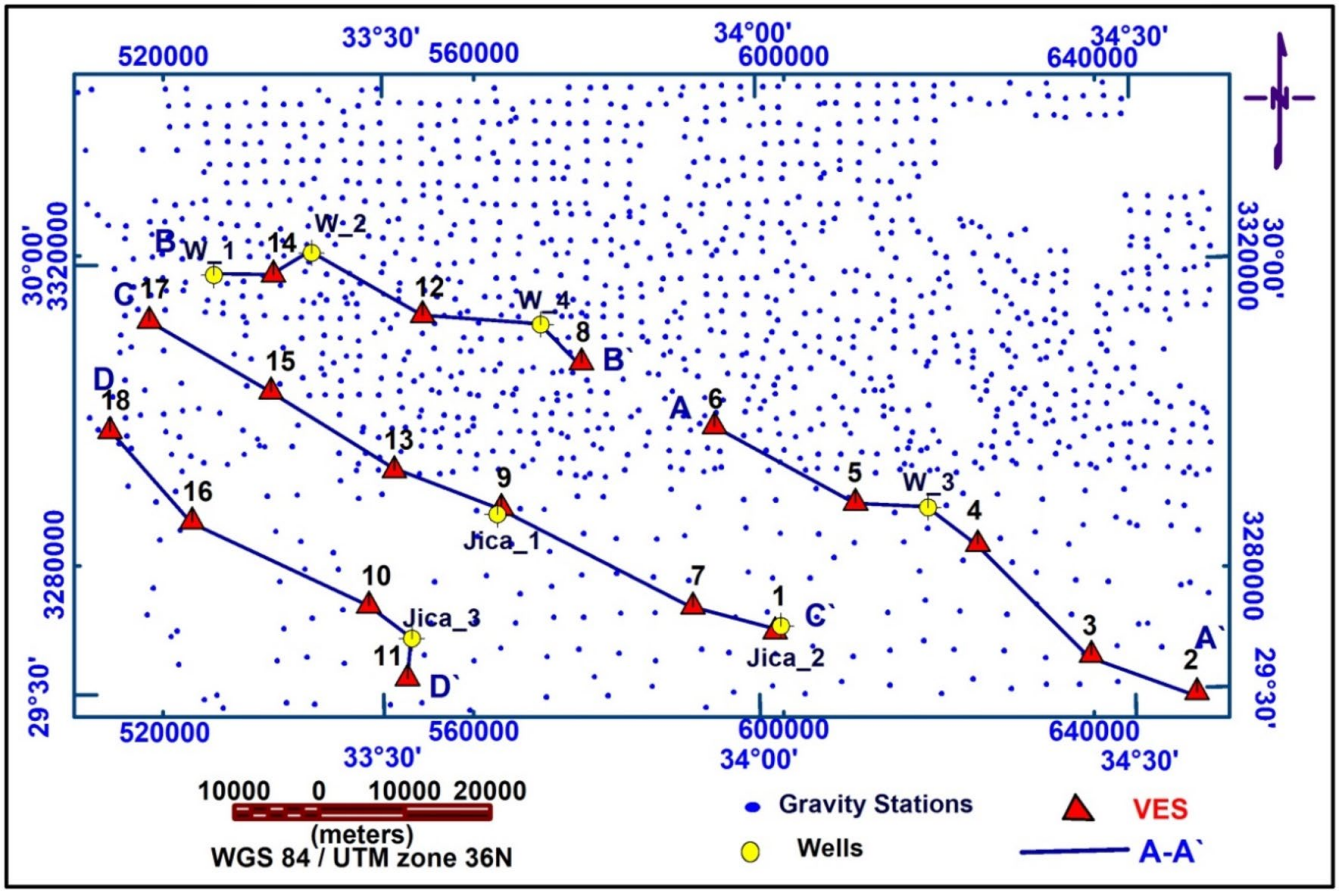
Locations of station points for measurements of gravity, locations of wells, VESes, and cross sections of electrical resistivity.

**Figure 7:**
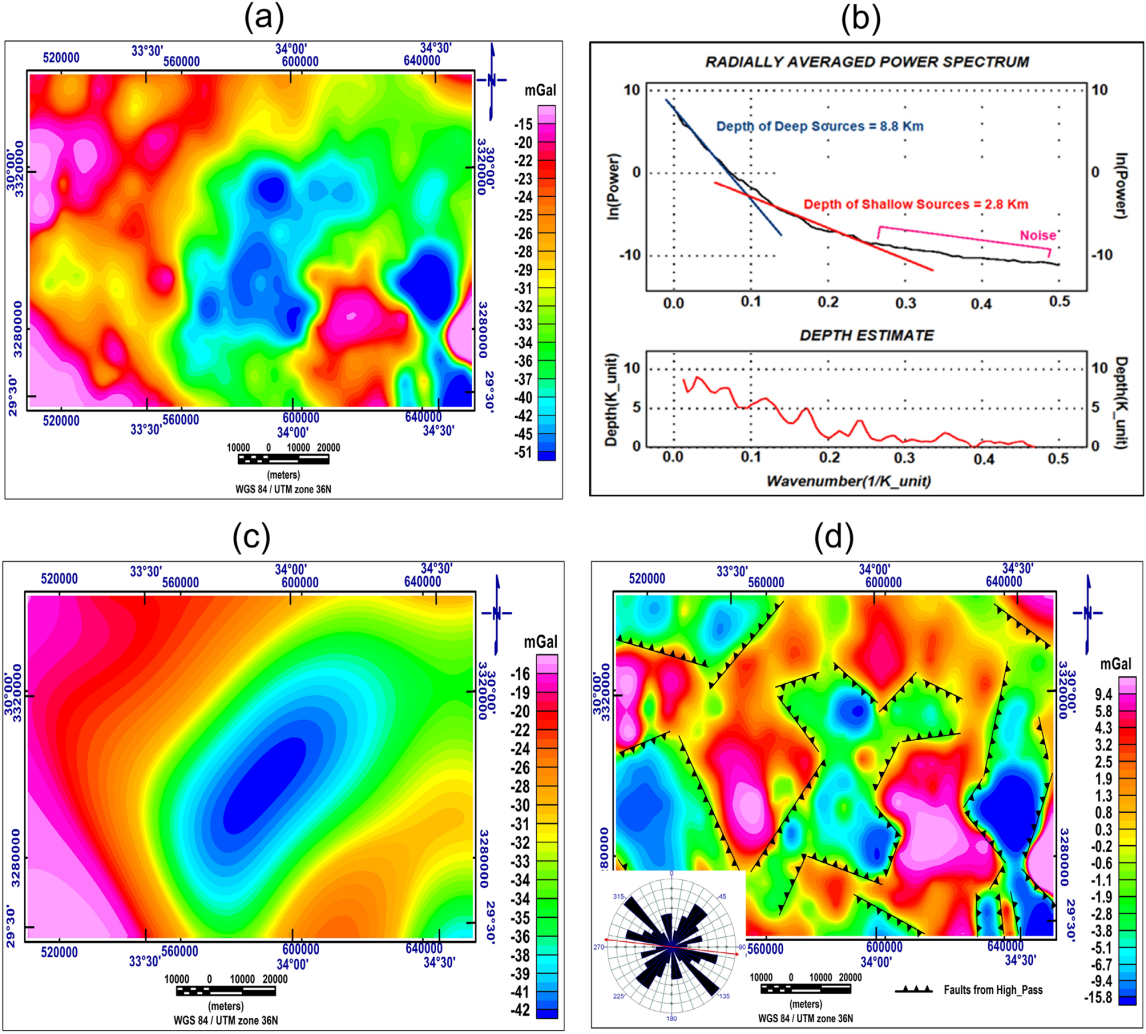
(**a**) Bouguer anomaly map, (**b**) power spectrum curve, (**c**) low pass (regional) gravity anomaly map, and (**d**) Fault elements dissecting residual gravity anomaly map with rose diagram.

The Egyptian Geological Survey has acquired the One thousand two hundred and two (1202) gravity stations (Fig. 6)^54^. To make the Bouguer anomaly map (Fig. 7a) for the area, gridded data and contoured by^55^ using the minimum curvature option. There is a maximum gravity anomaly field in this investigation area (− 15 mGal) at the NW, SW, and certain portions of the SE, and a minimum amount (− 51 mGal) at the SE, E, and central part of the research area, according to an examination for the map Bouguer anomaly. Uplift of denser basement rock is primarily responsible for the high gravity anomaly, whereas sedimentary basins are indicated by lower gravity readings.

### Gravity separations and structural

Separating anomalies of various wavelengths from one another is a primary objective of the filtering techniques. Native anomalies caused by shallow sources correspond to short wavelength or high-frequency anomalies, whereas regional variations caused by deep sources correspond to lengthy wavelength or lower frequency anomalies. Using wavenumber (0.018 cycles/K-unit) from the power spectrum curve, Bouguer anomaly mapping^55^, which was previously gridded and contoured, is dynamically divided into regional and residual components (Fig. 7b). A low-pass (regional) gravity filter accepts long wavelengths while rejecting any shorter wavelengths than the cut-off wave number. After the regional influence is affected, the gravity field's distribution at shallower depths is shown on the high-pass gravity anomaly map (Fig. 7c,d). Mapping the research area's residual gravity anomaly field shows the greatest values (9 mGal) in the NW, S, and SE and some areas of the research area, as well as minimum values (− 16 mGal) in the SE, NW, and SW and some study area portions. Lower gravity values reflect sedimentary basins, while the positive gravity anomaly is mostly caused by the uplift of high-density basement rock. High pass map interprets the research area's structural formations (Fig. 7d) to achieve the best resolution of the angle and lengths of the derived lineaments to aid in the identification of the main structural patterns in the area.

### VES data analysis and interpretation

**Figure 8:**
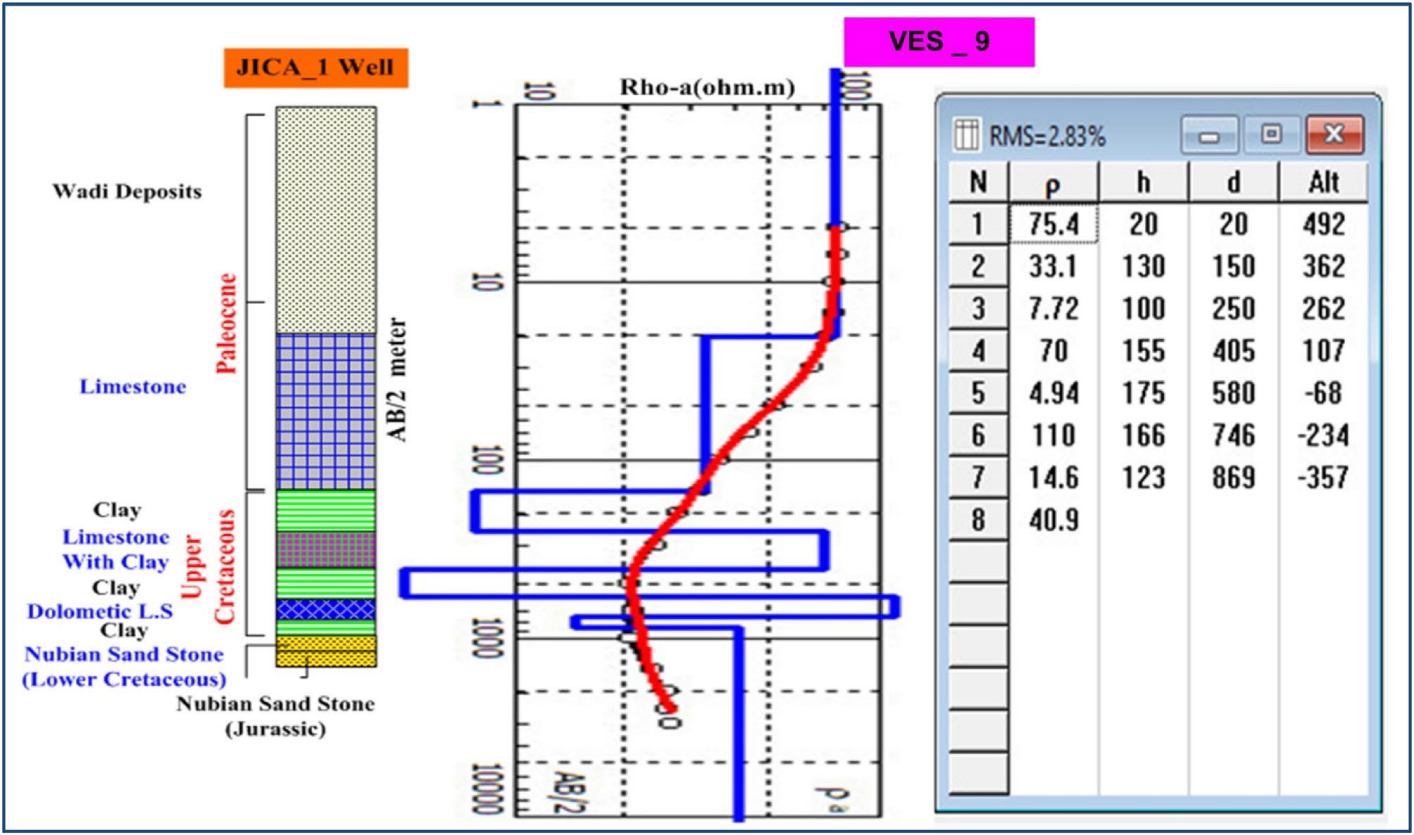
Calibration of the 1D model at VES-9 using the well JICA-1’s lithological log.

**Figure 9:**
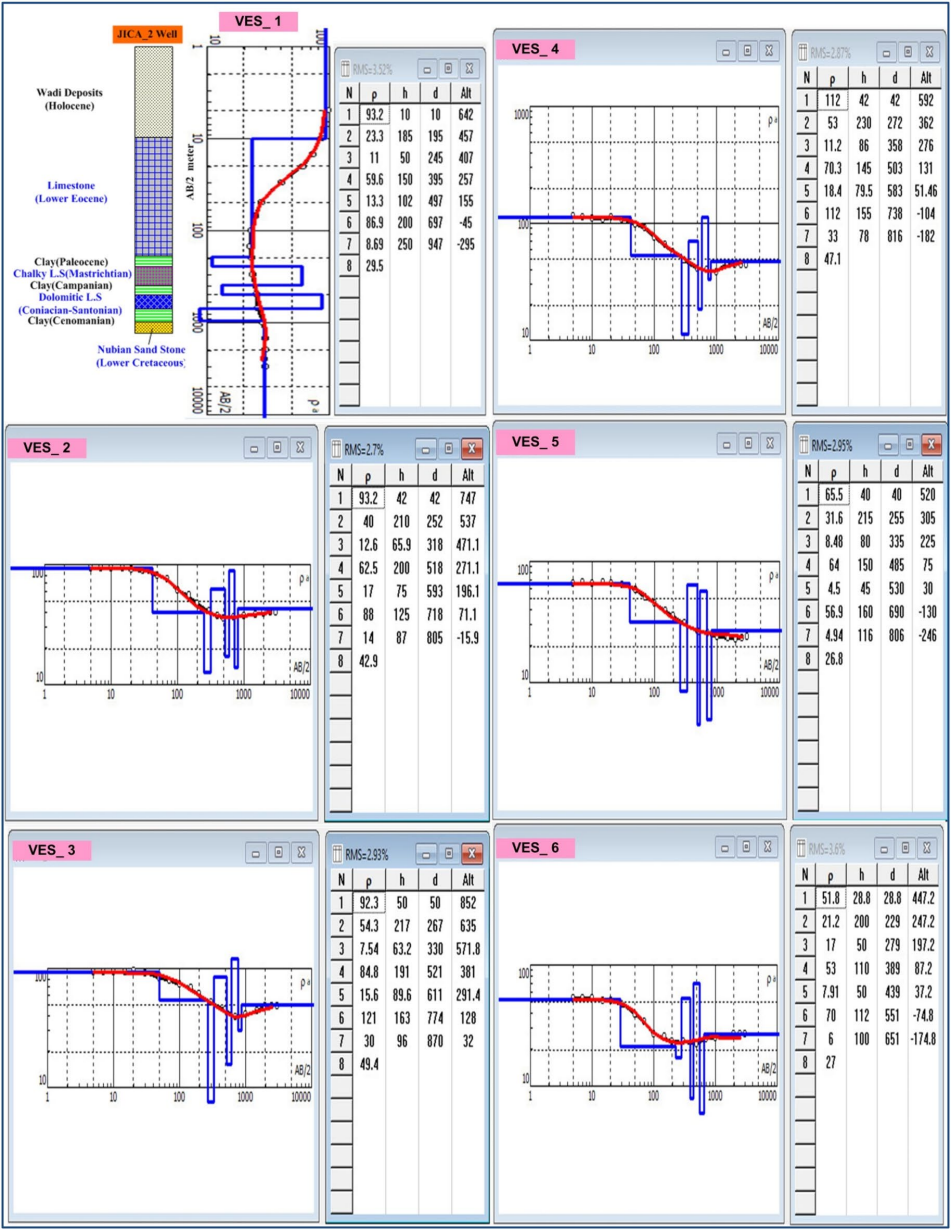
Interpretation of VES stations no. 1, 2, 3, 4, 5, and 6 (Using IPI2win software).

**Figure 10:**
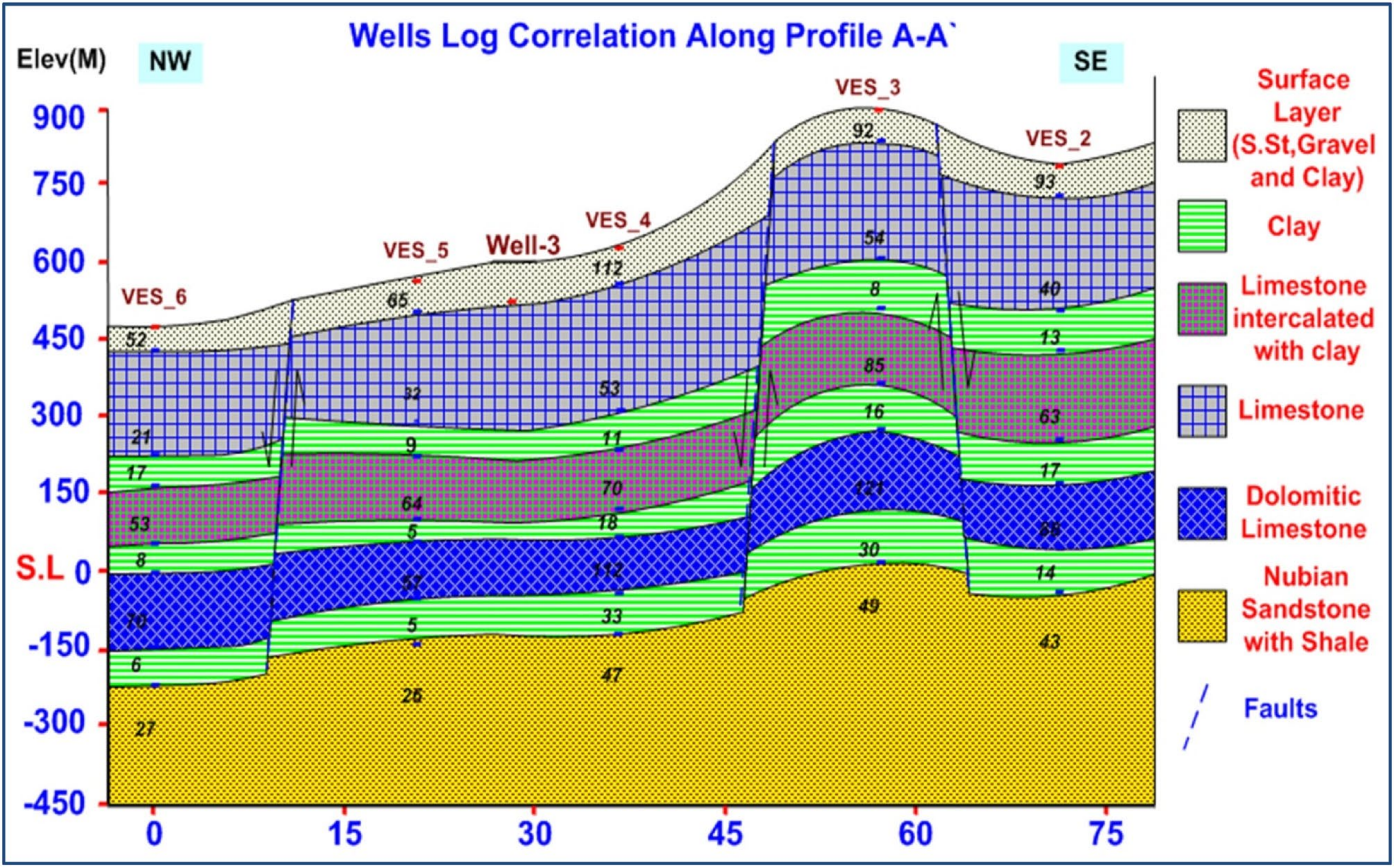
Geoelectrical cross-section along profiles A–A′

**Figure 11:**
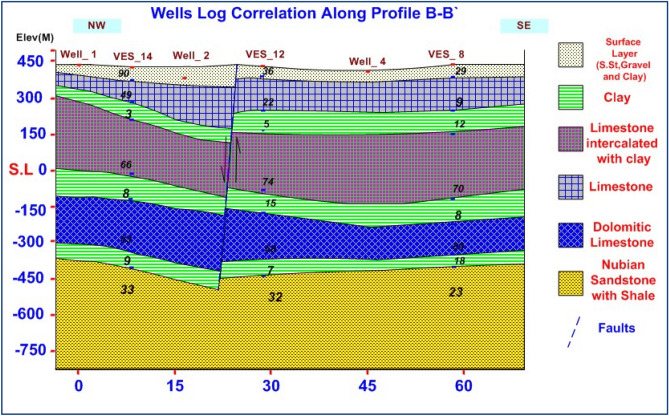
Geoelectrical cross-section along profiles B–B′

**Figure 12:**
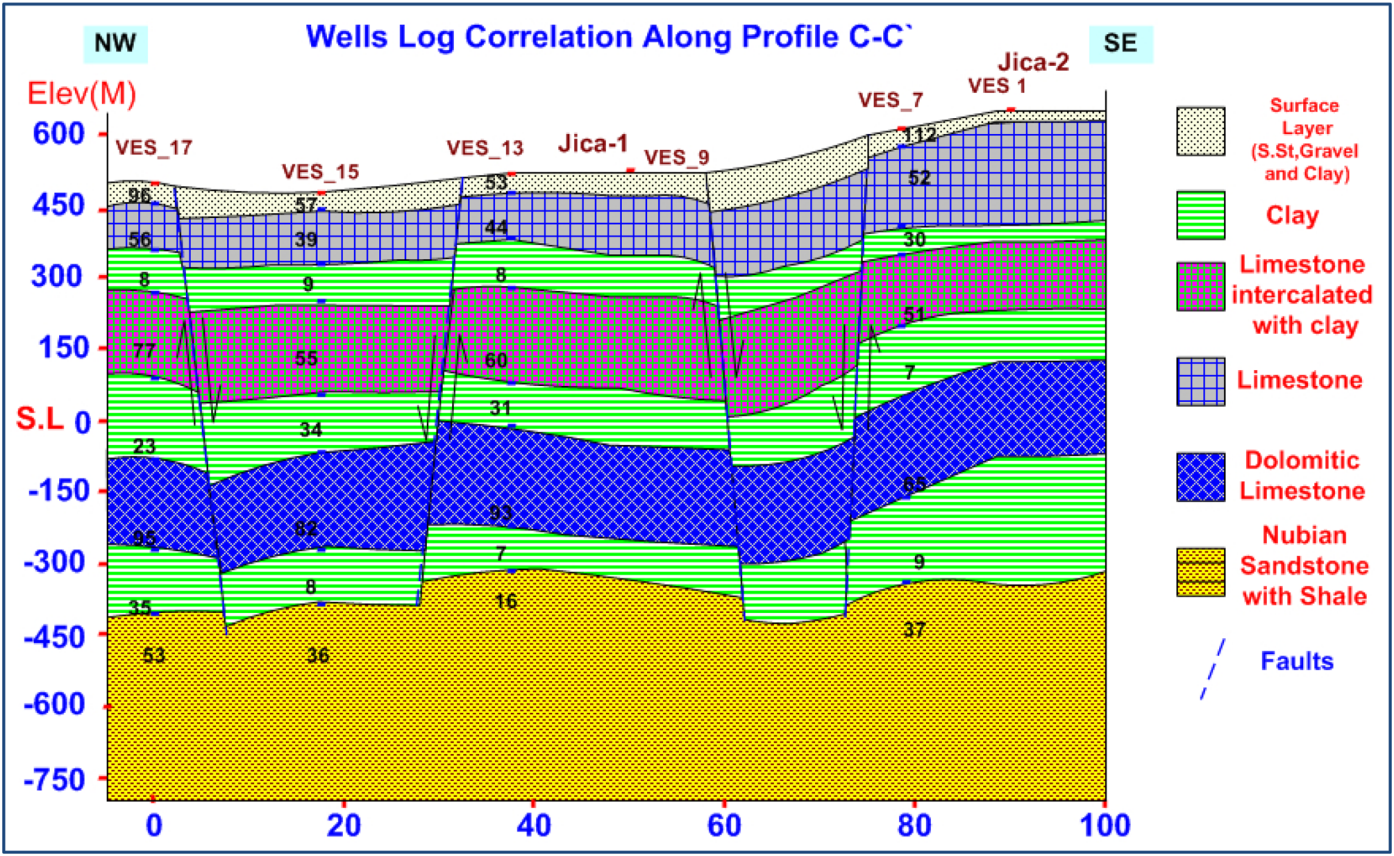
Geoelectrical cross-section along profiles C–C′

**Figure 13:**
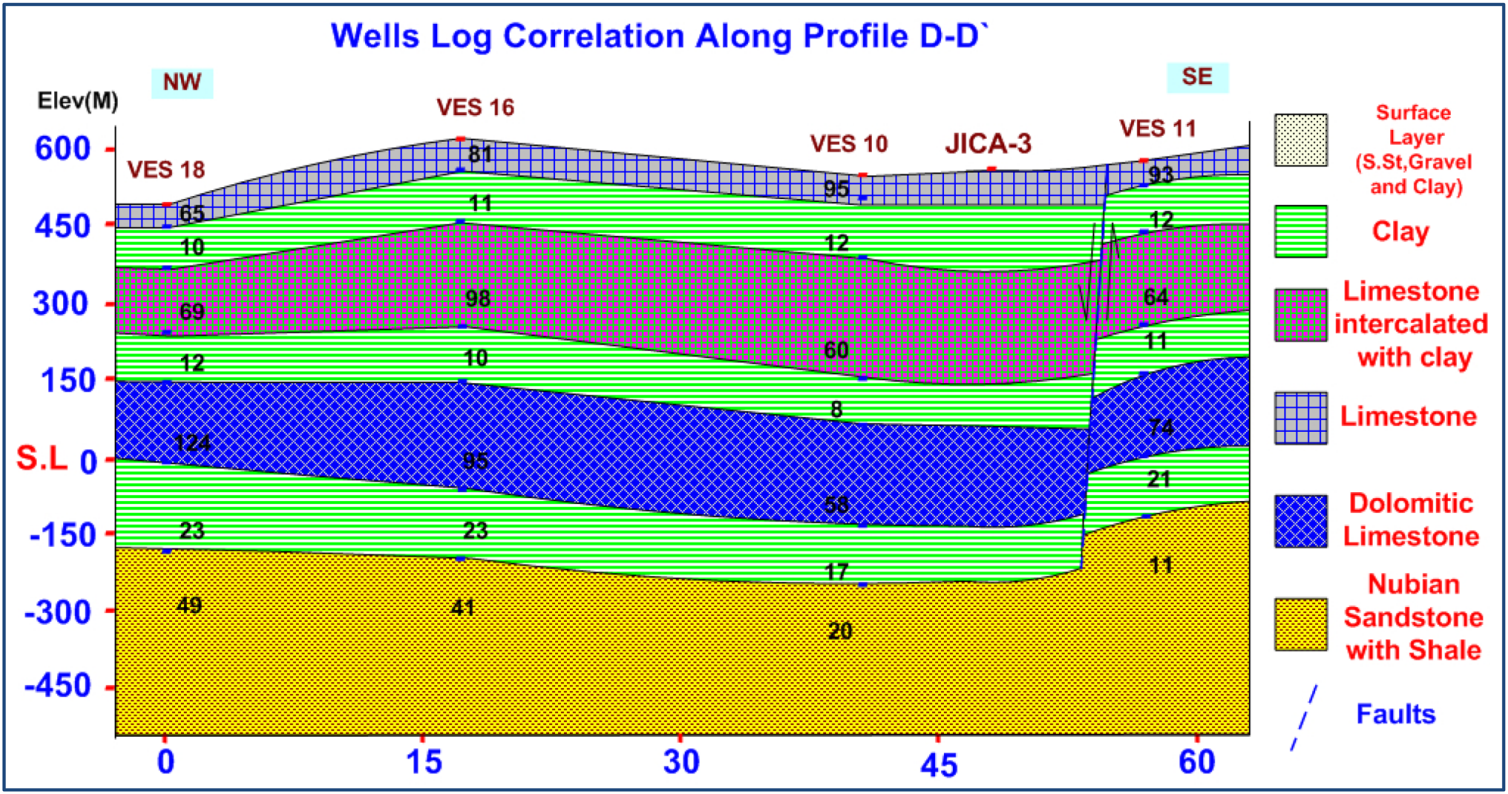
Geoelectrical cross-section along profiles D–D′

VES study determines geoelectrical layers' number, resistivity, and thickness beneath VES. Eighteen VES stations were used in the study (Fig. 6). It depicts a subset of the computed field curve, and Four profiles were inspected in Fig. 6. Two VES stations (1 and 9) were measured beside water wells to correlate with borehole data and provide them with geological and hydrogeological significance. In the VES measurements, the Schlumberger electrode design was employed with the greatest possible current electrode separation of 3000 m using a Syscal R2 resistivity meter. The tool measures resistance immediately and accurately. The exact locations and ground elevations were determined using a land topography survey and a GPS device. The VES-9, 1D model was calibrated using the lithology log from Well JICA-1 (Figs. 8 and 9). The calibration revealed that the VES-9 site had eight geoelectrical layers. VES field curve analysis uses calibration output as an indicator. Thus, the stratigraphic sequence of the geoelectrical succession was separated into eight main layers, which correspond to an age range from the Cretaceous to the Quaternary (Table 2). The distribution of horizontal and vertical subsurface resistivity can be seen in cross-sections of geoelectrical resistivity, which are established as vertical slices along the subsurface^56,57^. The obtainable multilayer models were used to build four 1-D geoelectrical cross-sections along (North West–South East) trending profiles (A–A′, B–B′, C–C′, and D–D′). The point of these cross-sections is a representation of the subsurface geoelectrical layers, the aquifer geometry, and the extension zone. According to resistivity, these cross-sections show both horizontal and vertical lithology variations. Figure 6 depicts the locations and directions of these profiles, which reveal the actual resistivities, thickness, and predicted lithology of the subsurface layers. The geoelectrical cross-sections show eight geoelectrical units (Figs. 10, 11, 12, and 13), which include all VESes and borehole data. The final unit of the section is the eighth geoelectrical unit. The top part of this layer consists of thick-bedded grained Nubian sandstones, which form the main freshwater aquifer in this research area and the aquifer from which the majority of wells produce, at depths between (640 and 950 m), but the lower surface is inaccessible due to the geometry of the electrode arrangement used. Also, the sections are intervened by a number of faults between some VESes and boreholes, which correspond with the residual gravity anomaly map. Considerable variations in the thickness and facies of some of the encountered units are observed.

**Table 2:** Describes the lithology of the geoelectric layers.

Layer	Resistivity (Ω.m)	Thickness (m)	Lithology	Age
1	29–112	25–90	Sandstone, gravel, and clay	Quaternary
2	29–95	35–250	Limestone	Upper Cretaceous
3	3–30	25–150	Clay	
4	51–98	100–315	Limestone intercalated with clay	
5	5–34	25–150	Clay	
6	57–124	100–200	Dolomitic limestone	
7	5–35	25–200	Clay	
8	11–53	1301–3127	Nubian sandstone with shale	Lower Cretaceous

### The interpreted subsurface maps

These maps will give a more detailed picture of the lateral distribution of resistivities and thicknesses of the detected geoelectrical units.

### True resistivity contour map

**Figure 14:**
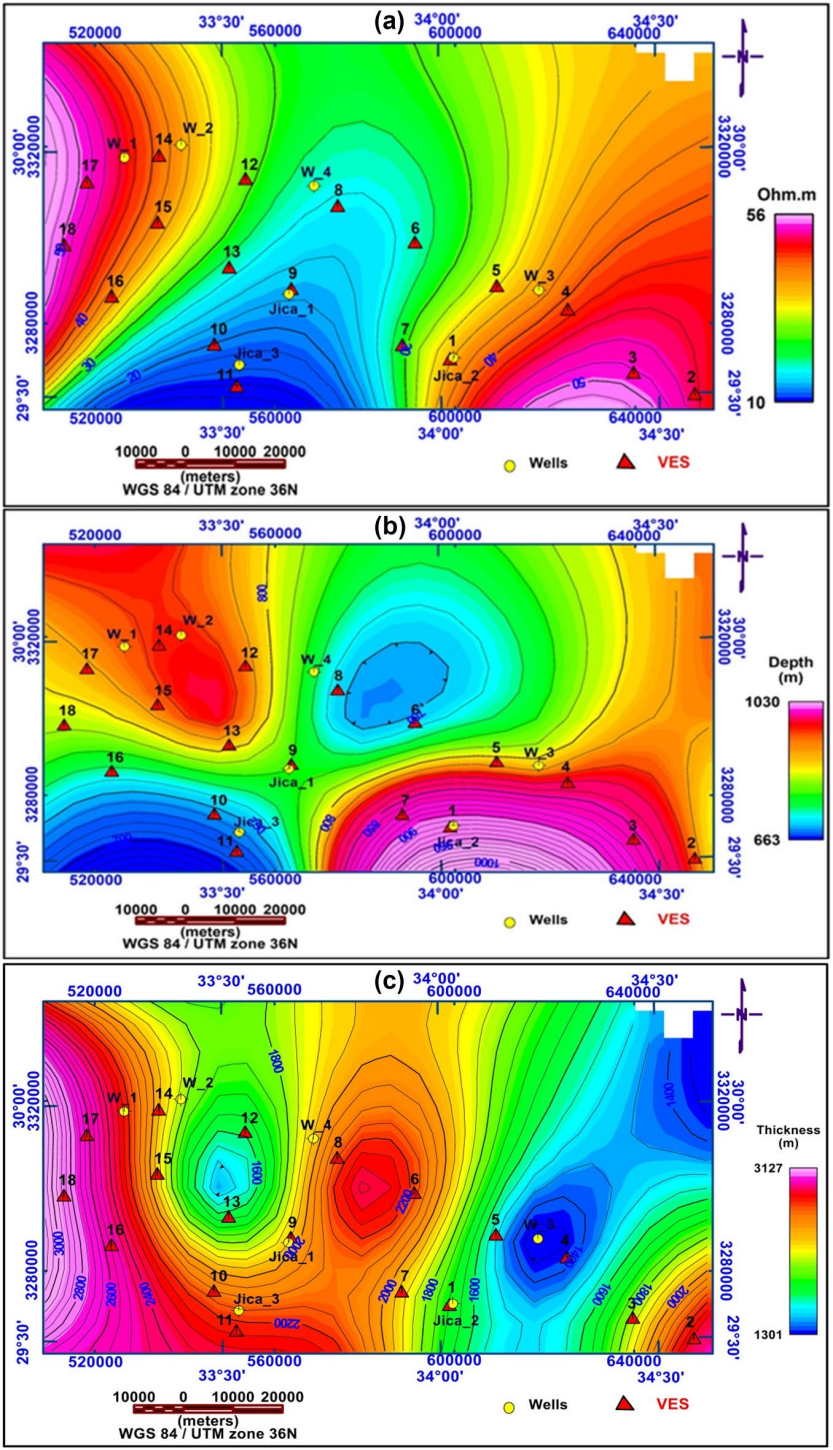
(**a**) True resistivity map for the eighth geoelectrical unit (Nubian S.S. aquifer), (**b**) Depth Contour Map, (**c**) Iso-Pach Contour Map.

The True Resistivity Contour map was created to show the variation in resistivity values for the Nubian sandstone layer (Fig. 14a), to differentiate the value of this layer from the water-bearing layer where the aquifer’s water quality is determined by resistivity values; the entire area has moderate resistivity, with just a little of the southern part being low resistive, according to the isoresistivity map for the eighth geoelectrical unit. The true resistivity varies between (15 Ω.m and 56 Ω.m), with an increase in resistivity primarily occurring in the NW and SE owing to a decrease in salinity. Wells are predominantly located in the Nubian Sandstone layer of the Lower Cretaceous Aquifer.

### Depth map

The purpose of constructing this map is to show the depth variations of the water-bearing unit to establish the localities for groundwater wells based on both bed thickness and depth to detect the best localities, which reduce the cost and increase the amount of the produced water.

The Depth Contour Map to the Eighth Geoelectrical Unit (Nubian S.S. aquifer) (Fig. 14b), reveals shallow depths in N-NE and SW zones of the research area (663–770 m), but deep depths in the northwestern and southeastern parts (780–1030 m).

### Iso-pach map

The Isopach map is constructed to indicate the variation of the thicknesses of eighth geoelectrical units. The thickness of the eighth geoelectrical unit in the study area is varied from 1301 to 3127 m (Fig. 14c). The lower surface of the eighth geoelectrical unit detects from the upper surface of the 2-D basement relief map according to^58^. The thickness increases mainly to the northern part, southern parts and a small part of the southeast in the study area. The lithology of this layer consists of sandstone and Sandy Shale for the Lower Cretaceous age.

### Priority map

**Figure 15:**
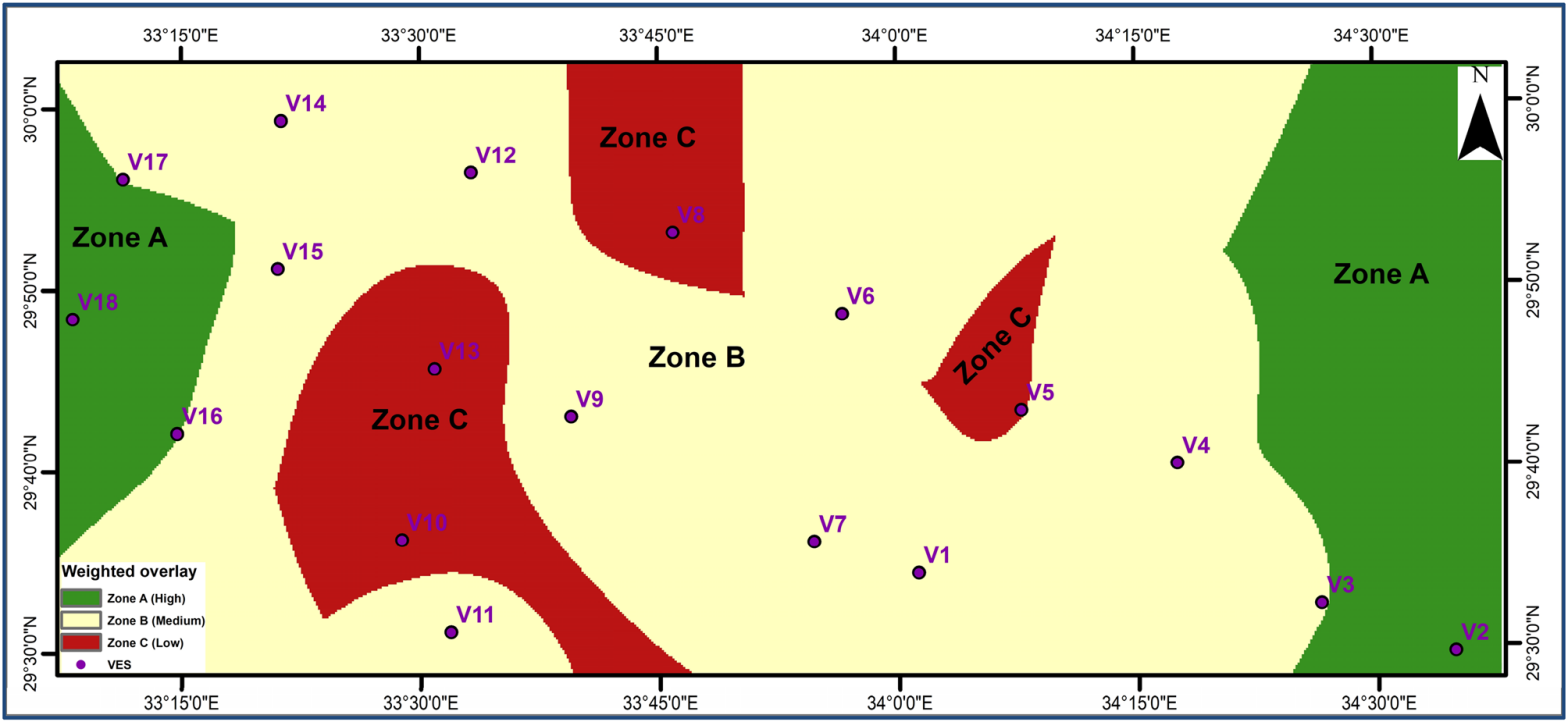
Priorities map for the upcoming drilling planned.

Figure 15 displays the priority map of drilling initiatives planned in the study area; Zone (A) offers favorable drilling opportunities in the freshwater aquifer (low depth, high resistivity values and large thickness). Zone (B) reflects medium depth, high resistivity values, and moderate thickness, and Zone (C) reflects large depth, low resistivity values, and Moderated thickness of the weighted overlay from Arc GIS.

### Results of integration and discussion

Geophysical field measurements such as VES surveys, land gravity data, borehole availability, and remote sensing were employed in the area under study to detect groundwater potential and locate drilling locations. The geological zone is separated into eight geoelectric layers that reflect Wadi deposits, limestone, clay, limestone intercalated with clay, dolomitic limestone, clay, and Nubian sandstone, following that basement complex, and can be utilized to describe the subsurface in the research area. Identification of groundwater potential requires a multi-factor problem, the use of GIS, both primary and secondary information, and four essential processes. First, identify the primary elements influencing groundwater potential. Rainfall as the primary source of water, slope, drainage density, lineaments, geology map, soil type map, and LULC are all elements that influence groundwater storage. Second, weights are assigned to these factors. Third, Utilize GIS tools to create raster maps with theme layers for relevant criteria. The GIS model generates significant groundwater potential maps in NW, NE, and N zones. Land gravity investigations have identified subsurface faults that have an impact on aquifer connections. The geoelectric method is one of the best approaches for exploring groundwater resources since it provides valuable data on the hydrological characteristics of shallow and deep aquifers. It is also advised that water collection structures be built in these regions. The second geoelectrical unit's first shallow aquifer has fractured limestone lithology ranging from 35 to 250 m. The second (main) deep aquifer is located in the eighth unit (Malhah Formation) and is composed of sandstone with shale and claystone intercalation. Drilled boreholes are ideally located around three zones (Priority Map) at VESs 16, 17, 18, 3, and 2, with depths ranging from 663 to 1030 m. Pumping tests can also be performed to estimate the hydraulic parameters for determining safe production rates. Surface geophysical surveys are recommended in these groundwater likelihood locations to realize the capacity of groundwater accessibility. Groundwater water storage is a valuable resource development method for Sinai, reducing losses from evaporation and natural disasters. This long-term plan should include filtering surface water and enhancing the groundwater resource. This study aims to provide hydrological, water resource, and environmental management data for informed planning and decision-making in promising hydrological locations in the basin.

## Conclusions and recommendations

Research focuses on multiple faults NE–SW and NW–SE, similar to Aqaba Gulf and Suez Gulf trends. These faults are interconnected, with the upward fault from the Nubian aquifer acting as a water pathway built on analyzing geophysical data, GIS, and remote sensing results. Groundwater movement, recharging, and discharge are facilitated by these fault and fracture systems. An eight-layer model reflecting Wadi deposits, limestone, Clay, limestone intercalated with clay, Clay, Dolomitic limestone, Clay, and Nubian sandstone, then a basement complex, can be used to explain the subsurface in the research area. The Nubian aquifer is found at depths 640–950 m. Groundwater quality is excellent, resistivity ranges varied from 15–56 Ω.m. The optimal drilling zone is in the NW and Eastern part of the area, a three-kilometer basin with high recharge potential. This area is regulated by fault systems and traps porous sediment accumulation.

Recommendations based on geophysical assessments and interpretations:

1. Three zones are prioritized on the groundwater priority map for the delta: the far NW and E parts is primary stream, the major northern stream, and the SE part.
2. The first zone (A) offer promising water drilling locations.
3. Most of Delta’s zones are moderately prospective and fall within the fourth priority group.

## Data Availability

The datasets generated and/or analyzed during the current study are not publicly available but are available from the corresponding author at reasonable request.
